# Global Brain Gene Expression Analysis Links Glutamatergic and GABAergic Alterations to Suicide and Major Depression

**DOI:** 10.1371/journal.pone.0006585

**Published:** 2009-08-11

**Authors:** Adolfo Sequeira, Firoza Mamdani, Carl Ernst, Marquis P. Vawter, William E. Bunney, Veronique Lebel, Sonia Rehal, Tim Klempan, Alain Gratton, Chawki Benkelfat, Guy A. Rouleau, Naguib Mechawar, Gustavo Turecki

**Affiliations:** 1 McGill Group for Suicide Studies, Douglas Mental Health University Institute, McGill University, Montreal, Quebec, Canada; 2 Royal Victoria Hospital, McGill University, Montreal, Quebec, Canada; 3 Ste Justine Hospital, Université de Montréal, Montreal, Quebec, Canada; 4 Department of Psychiatry and Human Behavior, School of Medicine, University of California Irvine, Irvine, California, United States of America; Universidade de Sao Paulo, Brazil

## Abstract

**Background:**

Most studies investigating the neurobiology of depression and suicide have focused on the serotonergic system. While it seems clear that serotonergic alterations play a role in the pathogenesis of these major public health problems, dysfunction in additional neurotransmitter systems and other molecular alterations may also be implicated. Microarray expression studies are excellent screening tools to generate hypotheses about additional molecular processes that may be at play. In this study we investigated brain regions that are known to be implicated in the neurobiology of suicide and major depression are likely to represent valid global molecular alterations.

**Methodology/Principal Findings:**

We performed gene expression analysis using the HG-U133AB chipset in 17 cortical and subcortical brain regions from suicides with and without major depression and controls. Total mRNA for microarray analysis was obtained from 663 brain samples isolated from 39 male subjects, including 26 suicide cases and 13 controls diagnosed by means of psychological autopsies. Independent brain samples from 34 subjects and animal studies were used to control for the potential confounding effects of comorbidity with alcohol. Using a Gene Ontology analysis as our starting point, we identified molecular pathways that may be involved in depression and suicide, and performed follow-up analyses on these possible targets. Methodology included gene expression measures from microarrays, Gene Score Resampling for global ontological profiling, and semi-quantitative RT-PCR. We observed the highest number of suicide specific alterations in prefrontal cortical areas and hippocampus. Our results revealed alterations of synaptic neurotransmission and intracellular signaling. Among these, Glutamatergic (GLU) and GABAergic related genes were globally altered. Semi-quantitative RT-PCR results investigating expression of GLU and GABA receptor subunit genes were consistent with microarray data.

**Conclusions/Significance:**

The observed results represent the first overview of global expression changes in brains of suicide victims with and without major depression and suggest a global brain alteration of GLU and GABA receptor subunit genes in these conditions.

## Introduction

Suicide accounts for almost 2% of the world's deaths, and in most developed countries it is the leading cause of death for males younger than 40 years of age [1]. Suicide is caused by a set of complex conditions and is frequently, but not exclusively, associated with depressive disorders. Although it is clear that these conditions are mediated by specific neurobiological processes [2], [3], the precise molecular alterations and the brain circuits involved in suicide and major depression remain largely unknown.

The suicide brain is believed to have a complex pattern of neurochemical alterations involving several neurotransmitter systems and different brain regions [4]. While most of the attention to date has focused on the possible dysregulation of the serotonergic system, and to a lesser extent, the noradrenergic neurotransmitter system [5]–[11], there is also evidence implicating other neurotransmitters, such as the dopaminergic [12]–[15], polyaminergic [16], glutamatergic [17]–[20] and GABAergic systems [4], [5], [21]–[23]. In addition, several studies have also investigated the role of signal transduction and other molecular systems [24]–[27]. Imaging studies of subjects with major depression and/or suicidal behavior using functional magnetic resonance and positron emission tomography have pointed to possible dysfunction of prefrontal neuronal circuits and subcortical areas of the brain, particularly some areas of the limbic system [28]–[34]. The complexity of neurotransmitter systems interacting in many distinct neuroanatomical regions underlines the need of a more comprehensive and inclusive approach monitoring alterations in different regions of the brain.

Microarray technology offers the possibility of parallel monitoring expression levels of several thousands to virtually all genes based on the hybridization of nucleotide probes mounted on high density arrays to a target nucleotide sequence [35], [36]. Recently this technology was implemented in psychiatry to study gene expression changes in postmortem brain tissue from psychiatric patients (for a review see [37]) and from suicide completers [16], [38], [39].

One of the major problems of experiments using dense microarrays is the level of multiple comparisons leading to false positive results. While different statistical approaches exist to correct for type I errors [40]–[42], independent replication, both internal and external, is the method of choice to determine the accuracy of results [43]. We hypothesized that biological processes that are globally altered across different brain regions believed to be implicated in the neurobiology of suicide and major depression are likely to represent valid global molecular alterations. Therefore, in this study, we conducted a global gene expression survey in 17 cortical and subcortical brain areas of male suicides with and without major depression versus matched psychiatrically normal controls aiming at the identification of molecular pathways that are differentially expressed, consistently, across those brain regions.

## Methods

### Subjects and diagnostic procedures

#### Quebec Suicide Brain Bank

Brain tissue was obtained from the Quebec Suicide Brain Bank. All samples used in the present study were from male subjects of French-Canadian origin, a homogeneous population with a well-known founder effect [44]. Cases and controls were group-matched for age and post-mortem interval. To be included in this study, suicides and controls had to die suddenly, with no medical or paramedic intervention, and with no prolonged agonal period. Brains were dissected at 4°C and snap-frozen in liquid nitrogen before storage at -80°C. Brain tissue was dissected and Brodmann areas (BA) identified in accordance with standard neuroanatomical definitions [45]. The anterior and posterior cingulate corresponded to the most anterior and the most posterior parts of the cingulate gyrus. In all cases, 1 cm^3^ human tissue blocks were paraffin-embedded, cryostat-sectioned, slide-mounted, and examined for any signs of disease by two independent pathologists in at least 3 different brain regions. No cases were excluded on this basis. This study was approved by our IRB and signed informed consent was obtained from next of kin.

All suicide and control subjects were psychiatrically diagnosed by means of psychological autopsies, which is a validated method to reconstruct psychiatric history by means of extensive proxy-based interviews, as outlined elsewhere [46]. In total, we analyzed 663 brain samples isolated from 39 subjects throughout the 17 regions, including suicides who died during an episode of major depression (SMD; N = 16); suicide victims with no history of major depression (S; N = 10); and matched psychiatrically normal controls (C; N = 13) who died suddenly from causes other than suicide and had no history of suicidal behavior. No other mood disorders were included in the present study. The vast majority of suicide completers from both the S and the SMD groups died by hanging. This is the most common method of suicide in Canada [46]. Controls died suddenly, without medical intervention by either accidents or myocardial infarctions. While this represents the total sample used in this study, there was some variability between regions following outlier exclusion (see below).

#### Controlling for alcohol confounding effects

To exclude the possible effect of alcohol on our positive findings, we followed up these results in an independent sample obtained from the University of California, Irvine (UCI) Brain Bank. This sample consisted of brain tissue from 13 male alcohol abusers and 21 controls, both groups psychiatrically normal otherwise. We investigated the dorsolateral prefrontal cortex (BA 9–46), a brain region that has been implicated in the etiology of alcoholism [47]. All subjects were clinically characterized by means of psychological autopsies and died suddenly without prolonged agonal state as described elsewhere [18]. All of the cases and none of the controls from the UCI sample had 6-month histories of alcohol abuse. Control for potential confounding effect of alcohol was also carried out by means of animal experiments as described below.

### RNA quality control and microarray experiments

All 663 RNA samples used in this study had a minimum A260/A280 ratio of>1.9 (mean = 2.03±0.14). The samples were further checked for evidence of degradation and integrity. Samples had a minimum 28S/18S ratio>1.6 and an average RIN of 7.14±0.85 (2100-Bioanalyzer, Agilent Technologies).

We used the HG-U133AB chipset, containing around 45,000 probe sets derived from approximately 33,000 human genes (http://www.affymetrix.com). Sample preparation and processing, hybridization to the Human Genome U133 Set, and normalization were performed as described in the Affymetrix GeneChip Expression Analysis Manual (Affymetrix, Santa Clara, CA) in collaboration with Gene Logic Inc (Gaithersburg, MD). The GeneChip IVT Express and the GeneChip® Hybridization, Wash, and Stain kits from Affymetrix were used for first and second cDNA synthesis, IVT/labeling and purification of aRNA, fragmentation and purification. GeneChip analysis was also performed based on the Affymetrix GeneChip Manual, with Microarray Analysis Suite (MAS) 5.1, Data Mining Tool (DMT) 2.0, and Microarray Database software. All of the genes represented on the GeneChip were globally normalized and scaled to a signal intensity of 100. Description of the Affymetrix normalization is available at the following site (http://www.affymetrix.com/support/technical/technotes/statistical_reference_guide.pdf).

Expression data was analyzed using Genesis 2.0 (GeneLogic Inc, Gaithersburg, MD) and AVADIS (Strand Genomics, Redwood City, CA). Several RNA integrity measures, in addition to 28S/18S ratios and RIN numbers, were used in this study to detect samples with poor RNA quality before final analysis: noise (RawQ), consistent number of genes detected as present across arrays, consistent scale factor, and consistent β-actin and GAPDH 5′/3′ signal ratios. Arrays with a significant deviation from the average RawQ, scale factor and 5′/3′ ratios were excluded. Problematic arrays were also identified using principal component analysis (PCA). Outlier subjects/arrays were excluded on a region specific basis, without any subject being excluded from all the regions. The data from this manuscript is available upon request.

### Semi-quantitative RT-PCR

For technical validation of differentially expressed genes, we performed semi-quantitative RT-PCR using RNA extracted from additional samples that were collected in each brain region from tissue adjacent to that used in the microarray expression study. All the subjects that passed quality control were also used in these experiments. Reverse transcription was performed in a total volume of 40 µl with 2 µg of total mRNA using M-MLV reverse transcriptase (Gibco, Burlington, Ontario) and oligo(dT)16 primers. PCR amplification was carried out using the Platinum Taq DNA Polymerase (Invitrogen, Carlsbad, CA), to determine the log linear phase of the amplification and to perform the semi-quantitative PCR. mRNA-specific primers, were designed using Primer3 (www-genome.wi.mit.edu/cgi-bin/primer/primer3_www.cgi) and their sequence is available upon request. Products were visualized using ethidium bromide staining after electrophoresis in a 3% agarose gel. Images were digitalized and analyzed using Gene Tools (Syngene, Cambridge). Experiments were carried in parallel in triplicate and β-actin was used as an internal control gene.

### Statistical analysis

The Microarray Suite software 5.1 (MAS 5.1) uses an algorithm that associates *P*-values to indicate statistical significance for gene expression detection and assign a Present, Marginal or Absent call. For each brain area, the list of genes were filtered prior to analysis such that only genes present (according to MAS 5.1) in at least 75 % of the subjects in at least one of the groups were included in our analyses. On average, 14,777 genes were analyzed per region across the 17 regions analyzed.

Gene expression values were floored to 1 and then log_2_-transformed. ANCOVAs were initially performed for each gene to identify statistically significant gene expression changes between the three groups, with substance abuse/dependence as a covariate. Statistically significant genes according to the ANCOVA were then subjected to a post-hoc t-test and fold-change analysis (FC) in order to identify pair-wise differences between the suicides with major depression (SMD), the suicides without major depression (S), and the controls (C). For a gene to be considered as differentially expressed, it had to have an ANCOVA *P*-value of less than or equal to 0.01 and a fold change of at least a 1.3 fold change in either direction. Post-hoc analyses were carried out using the Fisher protected LSD test with a *P*-value set at 0.01.

Cluster analysis was performed using average-linkage hierarchical cluster analysis with a correlation metric. Both expression patterns in individuals and genes were clustered. Principal component analysis (PCA) was performed based on the initial gene sets and on the selected genes (according to our significance criteria).

Functional ontological profiling of the expression changes was performed across all 17 regions using the Gene Score Resampling (GSR) method implemented in the ErmineJ software (version 2.1.8, Columbia University, NY) that examines the distributions of scores (FC or P-values) across the whole array [48]. This method compares the number of genes in a class ontology that show significant differential expression with the expected number of genes in that same class under the null hypothesis [48], eliminating the risk of finding false over-represented categories due to over-representation on the microarray chip. The parameters used were the following: Maximum gene set size: 300; Minimum gene set size: 5; with the mean of replicates, 10,000 iterations and full resampling. The rank and *P*-value computed by ErmineJ were used to calculate the most overrepresented ontologies across all regions. The distribution pattern of the ErmineJ calculated *P*-values in the different regions of the brain was examined by hierarchical clustering using AVADIS, with the normalized negative log of the *P*-values as the input. Further annotations were conducted using the Database for Annotation, Visualization and Integrated Discovery (DAVID) [49].

### Animal experiments

Adult male Sprague Dawley rats (Charles River, St. Constant, Québec) housed individually in clear Plexiglas cages (46 cm×18 cm×30 cm) on a 12-hr reverse-light cycle with food and water available *ad libitum* were used to study the effect of alcohol consumption on selected genes. All procedures were conducted in accordance with guidelines established by the Canadian Council on Animal Care.

For the acute ethanol (EtOH) administration, rats received a single injection of either vehicle (*n* = 5; 1 ml/kg ip) or EtOH (*n* = 5; 2.5 g/kg EtOH ip; 15% v/v EtOH in 0.9% saline) [50], [51]. The prefrontal cortex (PFC) was quickly dissected, flash frozen in isopentene and stored at −80°C until further analysis. For the chronic ethanol administration, food and water consumption were monitored for 3 days prior to treatment to ensure no differences in baseline consumption existed between the treatment groups. Rats were randomly assigned to one of 3 treatment conditions: water control (*n* = 5), sucrose control (*n* = 5; 10% sucrose solution) or EtOH (*n* = 5; 15% EtOH in a 10% sucrose solution). Once rats in the EtOH group readily drank the 10% sucrose solution (for 1 day) they were gradually habituated to the 15% EtOH solution [52]_._ EtOH rats received 5% EtOH in 10 % sucrose for 1 day, followed by 10% EtOH in 10% sucrose for 2 days. The solution was then changed to the 15% EtOH in 10% sucrose. Chronic EtOH treatment persisted for 28 days once the rats had access to the 15% EtOH solution. Twenty-nine days after the 15% EtOH treatment began, rats were sacrificed prior to lights off. Brains were removed and PFC was quickly dissected, flash frozen in isopentane, and stored at −80°C until subsequent analysis.

## Results

### Global analysis

Demographic and clinical characteristics of the subjects included in this study are shown in Table 1. No significant differences were observed between the groups for different demographic measures such as age (mean±sd: C = 35±11; S = 34±9; SMD = 37±13;), post-mortem interval (mean±sd: C = 24±6; S = 29±15; SMD = 25±7), or brain tissue pH (mean±standard deviation: C = 6.44±0.26; S = 6.32±0.27; SMD = 6.55±0.32). Furthermore, no significant correlation was observed in our sample as a whole between quality control parameters such as noise (RawQ), number of genes detected as present across arrays, scale factor, β-actin and GAPDH 5′/3′ (data not shown). This suggests that RNA quality from our tissue was acceptable, probably reflecting our brain recruitment procedures, which are limited to sudden death without medical intervention, prolonged agonal periods or extended PMI. All subjects in this study underwent toxicological screens and we detected only one subject with an SSRI in his blood, suggesting that medication is not a confounding factor in this study (Table 1).

**Table 1:** Demographic, clinical and toxicological characteristics of the subjects included in the study.

Group	Age	PMI	Cause of death	DSM-IV (six months diagnosis)	Toxicology screening
C	51	15	Motor vehicle accident	Alcohol dependence	Alcohol
C	31	24	Cardiac arrest	Alcohol dependence	
C	19	32	Motor vehicle accident		
C	47	12	Cardiac arrest	Alcohol abuse	
C	30	30	Cardiac arrest		
C	28	27	Motor vehicle accident		
C	41	24	Myocardial Infarction		
C	31	29.5	Motor vehicle accident		
C	46	19.5	Myocardial Infarction		
C	21	24	Cardiac arrest		
C	27	20.5	Cardiac arrest		
C	32	26.5	Cardiac arrest	Cannabis abuse	
C	55	24	Motor vehicle accident		
S	38	23	Hanging	Alcohol dependence, cocaine dependence	Alcohol
S	21	21	Asphyxiation	OCD, Alcohol dependence	Alcohol
S	31	32.5	Hanging		
S	29	26.5	Hanging		
S	33	18	Hanging		
S	26	69	Hanging		
S	30	27	Stabbing	Paranoid schizophrenia	
S	36	25	Hanging		
S	51	21	Self inflicted gun shot	Alcohol dependence	
S	42	27	Carbon monoxide		
SMD	28	20	Hanging	MDD, alcohol dependence	Alcohol
SMD	22	11.5	Hanging	MDD, alcohol dependence	Alcohol, cocaine
SMD	53	14	Carbon monoxide	MDD	
SMD	26	34	Hanging	MDD	Cocaine
SMD	40	23	Hanging	MDD, alcohol dependence	
SMD	19	29.5	Hanging	MDD	
SMD	53	29	Hanging	MDD, alcohol dependence	
SMD	42	21	Drowning	MDD	SSRI
SMD	45	20.5	Self inflicted gun shot	MDD, pathological gambling	
SMD	35	31	Hanging	MDD, alcohol dependence	
SMD	39	25.5	Hanging	MDD	
SMD	49	32	Hanging	MDD, alcohol abuse	
SMD	40	22	Hanging	MDD	
SMD	53	33.5	Hanging	MDD	
SMD	18	27	Carbon monoxide	MDD	
SMD	22	20	Hanging	MDD	

C = control, S = suicide, SMD = suicide with major depression, MDD = major depressive disorder, SSRI = Selective serotonin reuptake inhibitor.

**Figure 1:**
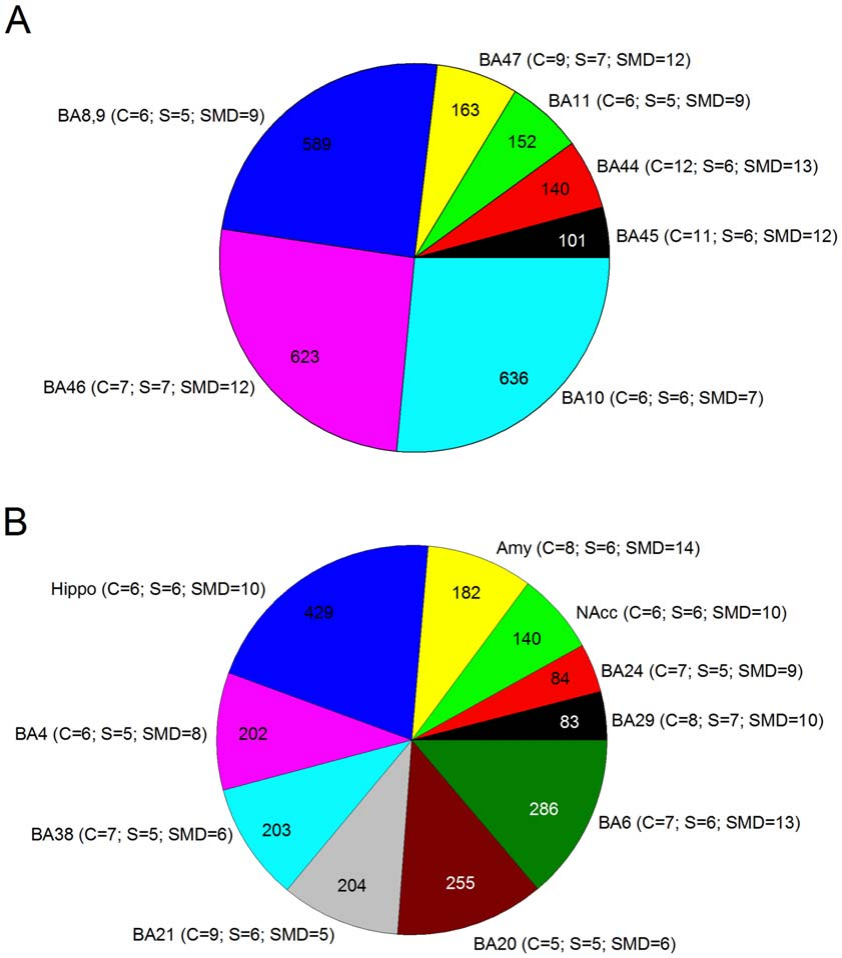
Pie charts representing the distribution of the total number of differentially expressed genes in (A) prefrontal cortical areas and in (B) other cortical and subcortical brain areas. The number of chips per group that passed quality control assessment is also given.

Overall, 251,206 probe sets passed the initial filtering criteria and were included in the analysis across the 17 regions with an average of around 15,000 probe sets per region. A summary of the analyzed and the differentially expressed probe sets per region and per group comparison, controlling for the possible effect of substance abuse/dependence, are shown in Table 2. Figure 1 provides the distribution of the total number of differentially expressed genes in prefrontal and subcortical brain areas. The region with the least probe sets analyzed was BA10 with 11,935 and the one with the most was BA45 with 15,886 probe sets. A total of 5,868 probe sets were significantly altered with the ANCOVA at the *P*≤0.01 level. Of these, 4,472 probe sets were differentially expressed across the regions after the Fisher protected LSD tests and fold-change (FC) filtering. These 4,472 probe sets were annotated to 3320 unique genes using DAVID. There was substantial variation between regions in terms of total number of differentially expressed genes, ranging from 83 in BA29 to 636 in BA10. In addition to BA10, the region with the second largest number of differentially expressed genes was BA46 with 626 genes. BA46 and BA10 are two prefrontal cortex regions which are anatomically close and have previously been associated with both suicidal behaviors and major depression. On the other hand, two limbic regions located in the cingulate cortex, BA24 and BA29, had the least number of differentially expressed genes, 84 and 83 respectively.

**Table 2:** Summary of the differential expression analysis in 17 brain areas of controls and suicides with and without major depression.

Brain Region	Genes analyzed	Genes significant	C-SMD	C-S	SMD-S
BA4 (C = 6; S = 5; SMD = 8)	14632	202	128 (66 up; 62 down)	55 (28 up; 27 down)	62 (35 up; 27 down)
BA6 (C = 7; S = 6; SMD = 13)	15266	286	216 (105 up; 111 down)	76 (31 up; 45 down)	46 (20 up; 26 down)
BA8,9 (C = 6; S = 5; SMD = 9)	14854	589	73 (33 up; 40 down)	302 (71 up; 231 down)	411 (147 up; 264 down)
BA10 (C = 6; S = 6; SMD = 7)	11935	636	604 (436 up; 168 down)	41 (30 up; 11 down)	69 (31 up; 38 down)
BA11 (C = 6; S = 5; SMD = 9)	14410	152	85 (19 up; 66 down)	33 (11 up; 22 down)	77 (50 up; 27 down)
BA20 (C = 5; S = 5; SMD = 6)	13944	255	77 (36 up; 41 down)	68 (21 up; 47 down)	173 (50 up; 123 down)
BA21 (C = 9; S = 6; SMD = 5)	14129	204	161 (42 up; 119 down)	20 (8 up; 12 down)	64 (37 up; 27 down)
BA38 (C = 7; S = 5; SMD = 6)	14395	182	65 (37 up; 28 down)	102 (58 up; 44 down)	64 (16 up; 48 down)
BA24 (C = 7; S = 5; SMD = 9)	15243	84	52 (27 up; 25 down)	20 (5 up; 15 down)	27 (15 up; 12 down)
BA29 (C = 8; S = 7; SMD = 10)	15032	83	19 (7 up; 12 down)	40 (13 up; 27 down)	40 (24 up; 16 down)
Amy (C = 8; S = 6; SMD = 14)	15007	153	95 (56 up; 39 down)	47 (22 up; 25 down)	35 (25 up; 10 down)
Hippo (C = 6; S = 6; SMD = 10)	14495	426	34 (16 up; 18 down)	118 (60 up; 58 down)	359 (196 up; 163 down)
NAcc (C = 6; S = 6; SMD = 10)	15232	140	22 (8 up; 14 down)	60 (10 up; 50 down)	91 (11 up; 80 down)
BA44 (C = 12; S = 6; SMD = 13)	15788	140	88 (32 up; 56 down)	24 (15 up; 9 down)	48 (30 up; 18 down)
BA45 (C = 11; S = 6; SMD = 12)	15886	101	60 (13 up; 47 down)	21 (8 up; 13 down)	27 (17 up; 10 down)
BA46 (C = 7; S = 7; SMD = 12)	15655	622	140 (37 up; 103 down)	192 (148 up; 44 down)	470 (373 up; 97 down)
BA47 (C = 9; S = 7; SMD = 12)	15303	163	102 (48 up; 54 down)	29 (13 up; 16 down)	51 (29 up; 22 down)
Total	251206	4472			

C = control, S = suicide, SMD = suicide with major depression, Amy = amygdala, Hippo = hippocampus, NAcc = nucleus accumbens. Information on Brodmann areas is provided elsewhere^93–94^. The number of genes analyzed corresponds to the number of genes considered as “Present” by the detection algorithm (MAS 5.1) in at least 75% of subjects in at least one of the groups. The number of genes reported as significant is that obtained in an ANCOVA model which included substance abuse/dependence as a covariate.

### Functional profiling

**Figure 2:**
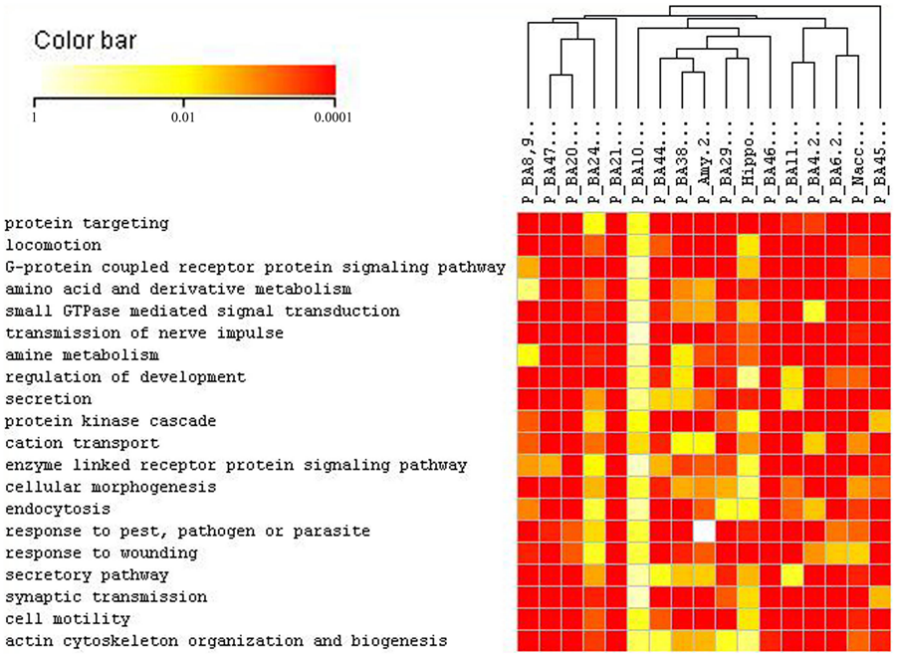
Clustered image map (CIM) of the hierarchical cluster analysis of the distribution pattern of ErmineJ calculated *P*-values of the 20 top overrepresented ontologies across all the regions studied. Both ontological categories and the regions were clustered. The color and intensity indicate level of significance: red spectrum colors indicate very highly significant gene ontologies (0.001), yellow colors indicate highly significant gene ontologies (0.01) and white colors represent no significance.

In order to identify altered functional pathways across all the regions investigated in this study, we initially used ErmineJ [48] to generate a list of overrepresented gene ontologies in each of the 17 brain regions independently and then the resulting significant ontologies were compiled to reflect the overlap of global ontologies. Subsequently, hierarchical clustering analyses were carried out to identify those biological processes that were commonly altered across all brain regions, Figure 2 shows the 20 top overrepresented ontologies. We then focused on the 10 most commonly overrepresented ontologies based on rankings and P-values in the 17 brain areas from the GSR analysis in ErmineJ. In order, from most to least commonly represented, these were signal transduction, intracellular signaling cascade, cell organization and biogenesis, protein localization, protein transport, establishment of protein localization, transmission of nerve impulse, small GTPase mediated signal transduction, synaptic transmission and vesicle-mediated transport. The corresponding probe sets belonging to these 10 globally overrepresented ontologies were further annotated using DAVID, resulting in the identification of 568 unique genes. As shown in Table 3, the majority of these genes corresponded to genes implicated in cell communication processes and related subcategories of functions such as intracellular signaling cascade, signal transduction, transmission of nerve impulse, and more specifically, synaptic transmission.

**Table 3:** The 568 unique genes identified in our database of differentially expressed genes were regrouped using DAVID according to their function.

Category	Term	Count	%	P-value
Biological Process	signal transduction	237	41.73%	2.57E-37
Biological Process	intracellular signaling cascade	138	24.30%	2.80E-42
Biological Process	cell organization and biogenesis	132	23.24%	1.38E-24
Biological Process	protein localization	79	13.91%	2.03E-25
Biological Process	protein transport	77	13.56%	8.29E-26
Biological Process	establishment of protein localization	77	13.56%	6.87E-25
Biological Process	transmission of nerve impulse	61	10.98%	6.73E-25
Biological Process	small GTPase mediated signal transduction	59	10.39%	1.54E-29
Biological Process	synaptic transmission	57	10.03%	2.50E-22
Biological Process	vesicle-mediated transport	55	9.68%	3.12E-20

As intracellular signaling cascade, signal transduction and transmission of nerve impulse are parent nodes related to synaptic transmission, a more specialized molecular function that is of particular interest to the neurobiological investigation of major depression and suicide, we explored more specifically the genes related to synaptic transmission. A total of 57 genes corresponded to this category (Table 4) and consisted of several pre-synaptic proteins (SYN2, SYPL1, SNAP25, SYT1, SYT5, SNPH) and signal transduction genes such as the mitogen-activated protein kinase 1 (MAPK1) and the 2′,3′-cyclic nucleotide 3′ phosphodiesterase (CNP). However, it was remarkable that a large proportion of these 57 genes (22 out of 57 or 38.6%) corresponded to genes implicated in GABAergic or glutamatergic neurotransmission or in the transport of these neurotransmitters (Table 4). For the following analyses, we then also opted to further explore GABAergic and glutamatergic genes, because of prior reports to their implication in both major depression [22], [23], [53]–[55] and in suicide [17], [19], [20], [55], [56].

**Table 4:** Differentially expressed genes directly implicated in synaptic transmission as determined using DAVID (2006).

Name	Symbol	Cytoband	Entrez Gene
2′,3′-cyclic nucleotide 3′ phosphodiesterase	CNP	17q21	1267
4-aminobutyrate aminotransferase	ABAT	16p13.2	18
5-hydroxytryptamine (serotonin) receptor 2a	HTR2A	13q14–q21	3356
adenylate cyclase activating polypeptide 1 (pituitary) receptor type i	ADCYAP1R1	7p14	117
amphiphysin (stiff-man syndrome with breast cancer 128 kda autoantigen)	AMPH	7p14–p13	273
apolipoprotein e	TOMM40	19q13	10452
cocaine- and amphetamine-regulated transcript	CART	5q13.2	9607
cortistatin	APITD1	1p36.22	378708
discs, large (drosophila) homolog-associated protein 1	DLGAP1	18p11.3	9229
double c2-like domains, alpha	DOC2A	16p11.2	8448
drebrin 1	DBN1	5q35.3	1627
dystrobrevin, alpha	DTNA	18q12	1837
gaba(a) receptor-associated protein like 1	GABARAPL1	12p13.2	23710
gamma-aminobutyric acid (gaba) a receptor, alpha 1	GABRA1	5q34–q35	2554
gamma-aminobutyric acid (gaba) a receptor, alpha 4	GABRA4	4p12	2557
gamma-aminobutyric acid (gaba) a receptor, alpha 5	GABRA5	15q11.2–q12	2558
gamma-aminobutyric acid (gaba) a receptor, beta 1	GABRB1	4p12	2560
gamma-aminobutyric acid (gaba) a receptor, delta	GABRD	1p|1p36.3	2563
gamma-aminobutyric acid (GABA) A receptor, gamma 1	GABRG1	4p12	2565
gamma-aminobutyric acid (gaba) a receptor, gamma 1	GRIA2	4q32–q33	2891
gamma-aminobutyric acid (gaba) a receptor, gamma 2	GABRG2	5q31.1–q33.1	2566
gamma-aminobutyric acid (gaba) b receptor, 2	GABBR2	9q22.1–q22.3	9568
gamma-aminobutyric acid (gaba) receptor, rho 1	GABRR1	6q14–q21|6q13–q16.3	2569
glutamate dehydrogenase 1	GLUD1	10q23.3	2746
glutamate receptor, ionotrophic, ampa 3	GRIA3	Xq25–q26	2892
glutamate receptor, ionotropic, ampa 1	GRIA1	5q33|5q31.1	2890
glutamate receptor, ionotropic, ampa 2	GRIA2	4q32–q33	2891
glutamate receptor, ionotropic, kainate 1	GRIK1	21q22.11	2897
glutamate receptor, ionotropic, n-methyl d-aspartate 2a	GRIN2A	16p13.2	2903
glutamate receptor, metabotropic 3	GRM3	7q21.1–q21.2	2913
glutamate-ammonia ligase (glutamine synthetase)	GLUL	1q31	2752
gtp cyclohydrolase 1 (dopa-responsive dystonia)	GCH1	14q22.1–q22.2	2643
mitogen-activated protein kinase 1	MAPK1	22q11.2|22q11.21	5594
myelin basic protein	MBP	18q23	4155
myelin oligodendrocyte glycoprotein	MOG	6p22.1	4340
nad(p)h dehydrogenase, quinone 1	NQO1	16q22.1	1728
neuronal pentraxin ii	NPTX2	7q21.3–q22.1	4885
neuropeptide y	NPY	7p15.1	4852
pallidin homolog (mouse)	PLDN	15q21.1	26258
peripheral myelin protein 22	PMP22	17p12–p11.2	5376
phosphatidylinositol 4-kinase, catalytic, alpha polypeptide	PIK4CA	22q11.21	5297
piccolo (presynaptic cytomatrix protein)	PCLO	7q11.23–q21.3	27445
potassium large conductance calcium-activated channel, subfamily m, beta member 4	KCNMB4	12q	27345
potassium voltage-gated channel, kqt-like subfamily, member 2	KCNQ2	20q13.3	3785
rab14, member ras oncogene family	RAB14	9q32–q34.11	51552
s100 calcium binding protein, beta (neural)	S100B	21q22.3	6285
sodium channel, voltage-gated, type x, alpha	SCN10A	3p22–p21	6336
solute carrier family 1 (glial high affinity glutamate transporter), member 2	SLC1A2	11p13–p12	6506
solute carrier family 1 (glial high affinity glutamate transporter), member 3	SLC1A3	5p13	6507
solute carrier family 6 (neurotransmitter transporter, creatine), member 8	SLC6A8	Xq28	6535
solute carrier family 6 (neurotransmitter transporter, gaba), member 1	SLC6A1	3p25–p24	6529
synapsin ii	SYN2	3p25	6854
synaptophysin-like 1	SYPL1	7q22.2	6856
synaptosomal-associated protein, 25kda	SNAP25	20p12–p11.2	6616
synaptotagmin i	SYT1	12cen–q21	6857
synaptotagmin v	SYT5	19q|11p	6861
syntaphilin	SNPH	20p13	9751

### Pathways globally differentially expressed

In order to specifically explore GABAergic and glutamatergic genes that were differentially expressed across the different brain regions, we interrogated the list of 5,868 differentially expressed genes using the probe sets identified and annotated using DAVID.

#### GABAergic genes

**Figure 3:**
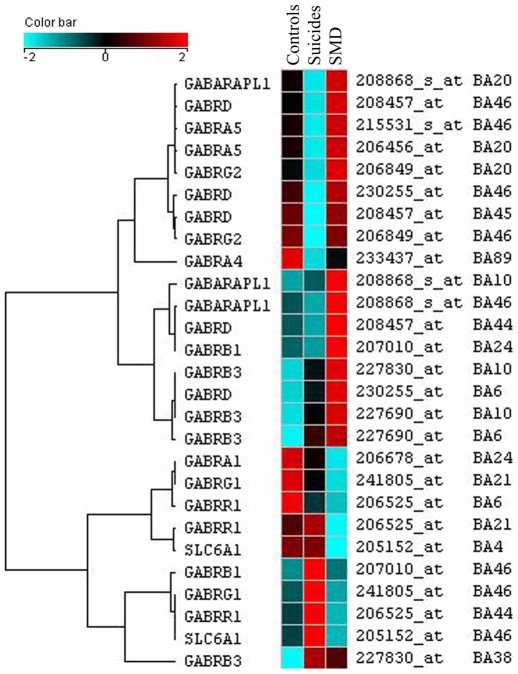
Clustered image map (CIM) of the hierarchical cluster analysis of the GABAergic differentially expressed subunit genes across the 17 regions investigated. The color and intensity indicate direction and level of change: blue spectrum colors indicate down-regulated expression, while red spectrum colors indicate up-regulated expression.

A total of 27 GABAergic-related probe sets were differentially expressed across the regions, many corresponding in fact to probe sets for the same genes as graphically represented in Figure 3. For instance, one gene, the Gamma-aminobutyric acid (GABA) A receptor, delta (GABRD) gene was differentially expressed in BA6, BA44, BA45, BA46 and the GABA(A) receptor-associated protein like 1 gene (GABARAPL1) was differentially expressed in BA10, BA20 and BA46 (Figure 3). Part of the ventrolateral prefrontal cortex, BA46 was of particular interest with a total of six GABAergic genes differentially expressed (GABARAPL1, GABRA5, GABRB1, GABRD, GABRG1, GABRG2). Also noteworthy, the majority of the differentially expressed GABAergic genes (19 out of 27) corresponded to different subunits of the GABA(A) receptor, particularly the alpha, beta, delta, gamma and rho subunits. As seen in Figure 3, a clear pattern of dysregulation was observed in terms of genes and regions implicated with a majority of GABAergic genes being up-regulated (red) among the suicides with major depression. For instance, in the hippocampus, all differentially expressed GABAergic genes were clearly up-regulated in suicides with major depression (GABARAPL1, GABARA4 and GABARB1) and with low expression among suicides without history of depressive disorders, suggesting a depression specific effect. Also seen in Figure 3, a total of 10 GABA(A) receptor beta probe sets were differentially expressed and were generally up-regulated among the depressed suicides. The same was observed for the GABA(A) receptor–associated protein like 1 (GABARAPL1) which was up-regulated in the depressed suicide group in BA10, BA20, BA46 and hippocampus. In summary, a striking number of probe sets corresponding to GABA(A) receptors or GABA(A) receptor-associated binding protein were altered between the three groups, with the majority being up-regulated among the suicides with major depression and having lower expression levels among the suicides without major depression or the controls, suggesting their role in molecular processes that may be more specific to the pathophysiology of major depressive disorder.

#### Glutamatergic genes

**Figure 4:**
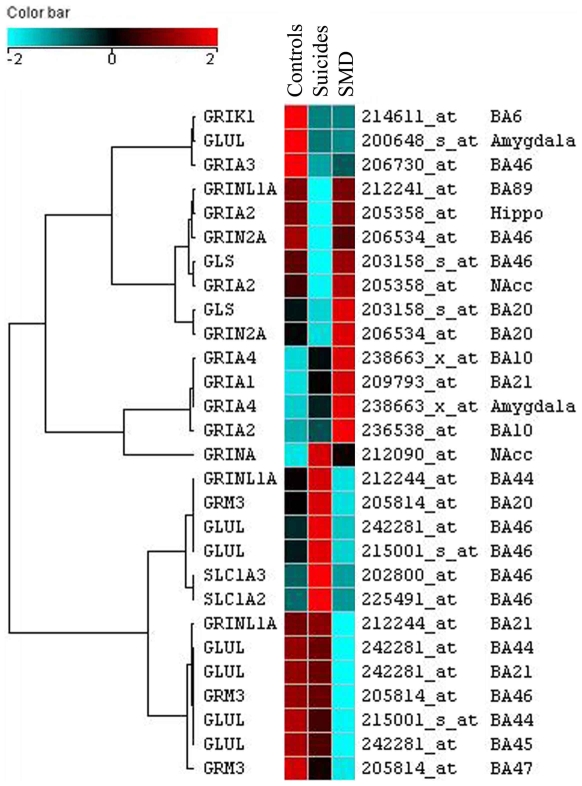
Clustered image map (CIM) of the hierarchical cluster analysis of the glutamatergic system differentially expressed genes across the 17 regions investigated. The color and intensity indicate direction and level of change: blue spectrum colors indicate down-regulated expression, while red spectrum colors indicate.

A total of 28 probe sets corresponding to genes implicated in glutamatergic neurotransmission were differentially expressed across the regions. A graphical representation of the gene expression changes between the three groups is shown in Figure 4. A good proportion of these probe sets (7) corresponded to the Glutamate-ammonia ligase (glutamine synthase) gene (GLUL) that codes for an enzyme implicated in glutamate recycling. GLUL probe sets were found consistently down-regulated among the depressed suicides in the prefrontal cortex (BA44 BA45, BA46) and the amygdala (Figure 4). Also of particular interest, 7 out of 28 probe sets (Figure 4) correspond to one of four subtypes (AMPA1, AMPA2, AMPA3, AMPA4) of the glutamate AMPA receptor that was differentially expressed in several brain cortical (BA10, BA21, BA46) and subcortical areas (hippocampus, nucleus accumbens, amygdala). A majority of the glutamatergic related probe sets correspond to ionotropic NMDA receptor subunits (GRINA, GRIN2A, GRINL1A) and AMPA (GRIA3,GRIA4, GRIA1, GRIA2) receptors with the later being consistently up-regulated among the suicides with major depression versus the controls or the suicides without history of major depression (Figure 4). Also noteworthy, the glutamate receptor metabotropic 3 (GRM3) was consistently down-regulated among the suicides with and without major depression in two areas of the prefrontal cortex BA46 and BA47 and in two areas of the parietal cortex BA38 and BA20 (Figure 4). In summary, we observed a global up-regulation of AMPA receptors and a global down-regulation of the GRM3 receptor and the glutamine synthase (GLUL) genes expression in the suicide with major depression group.

### Validation of microarray gene expression results

The differential expression of GABAergic and glutamatergic genes was further confirmed by means of semi-quantitative RT-PCR in additional samples from the same individuals that were immediately adjacent to those used for the microarray assays. We investigated only the brain areas where differential expression was observed in the microarray experiments (Table 5). Out of the 16 genes/regions tested, 15 showed the same expression direction (up-/-down regulation) in comparisons between groups as those observed in the microarray experiments, and 12 were also significantly differentially expressed in the semi-quantitative RT-PCR experiments (Table 5).

**Table 5:** Confirmation of the microarray results involving critical GABAergic and glutamatergic genes in major depression and suicide using independent adjacent samples from the same subjects/areas by semi-quantitative RT-PCR (SemiQ RT-PCR).

		SemiQ RT-PCR (Mean)				Affymetrix (Mean)			
Gene	Region	Control	Suicide	SMD	*P*	Control	Suicide	SMD	*P*
GABARAPL1	BA46	135.35	102.88	171.73	0.13	197.566	181.244	261.768	0.002
GABARD	BA45	70.96	49.53	69.86	0.04	265.784	190.645	273.365	0.002
GABARD	BA46	196.79	92.43	109.35	0.04	405.741	300.856	443.493	0.003
GABARG1	BA21	64.17	85.35	61.89	0.13	262.739	215.787	170.116	0.002
GABARG1	BA46	35.92	46.04	31.30	0.02	120.571	174.447	107.812	0.000
GABRG2	BA46	52.95	36.15	48.91	0.04	416.956	267.694	418.698	0.007
GABRR1	BA44	195.05	376.23	138.60	0.01	23.6967	35.345	19.1808	0.007
GLS	BA46	56.56	48.36	56.43	0.12	231.561	163.459	243.658	0.002
GLUL	Amy	93.46	76.52	68.49	0.02	789.678	438.535	422.579	0.002
GLUL	BA21	131.67	118.04	107.04	0.08	463.524	442.932	265.696	0.002
GLUL	BA45	142.64	128.99	108.66	0.02	338.71	307.803	197.303	0.002
GLUL	BA46	128.30	184.69	87.91	0.01	251.503	361.113	190.146	0.001
GRIA1	BA21	71.30	67.63	142.03	0.04	118.831	150.885	180.902	0.004
GRIA3	BA46	82.77	48.63	51.32	0.05	132.681	93.5443	100.471	0.006
GRM3	BA46	70.75	52.57	38.63	0.02	286.41	281.21	241.308	0.004
SLC6A1	BA4	150.61	156.09	140.69	0.58	648.13	648.05	478.61	0.002

Mean values per group are shown for the SemiQ RT-PCR experiment as well as for the MAS 5.1 normalized Affymetrix gene expression experiment.

### Controlling for potential confounding effect of alcohol

The possible effect of alcohol on gene expression was a potential confounder of our results, considering that a comorbid diagnosis of alcohol abuse/dependence was present in some of the subjects in the three groups. To exclude the potential confounding effect of alcohol on our results, we first conducted analyses of covariance (Table S1) investigating genes associated with substance use across all the brain regions. None of the GABAergic or glutamatergic genes differentially expressed in suicides with or without major depression were associated to substance use.

We also investigated whether or not these GABAergic or glutamatergic genes were associated with alcohol in an independent gene expression dataset from the UCI Brain Bank. To this end, we compared samples from the dorsolateral prefrontal cortex of 13 alcohol abusers to samples from the same brain region of 21 normal controls using the Affymetrix HG-U133 Plus 2 microarrays. Results are presented in Table S2 and suggest that none of the GABAergic or glutamatergic genes found to be globally differentially expressed in depression and suicide were significantly differentially expressed in alcoholics.

Finally, we studied the long- (one month) and short-term (5 days) effects of alcohol on GABARD, GABRG2, GLS, GLUL, GRIA1, GRIA3, GABARG1, GRM3, GABARAPL1, SLC6A1, and GABRR1 gene expression by semi-quantitative RT-PCR in rat prefrontal cortex. We found no significant differences for these genes in either chronic or acute alcohol treatments (Table S3).

## Discussion

In this study, gene expression was investigated using genome-wide microarrays in 17 brain areas thought to be involved in the neurobiology of suicide and major depression, comparing suicides with and without major depression to psychiatrically normal controls. This is, to our knowledge, the first large-scale brain expression study aiming at identifying global brain alterations associated with suicide and major depression. The extent of the expression changes varied considerably between the diverse brain areas investigated, with certain areas, such as those that comprise the prefrontal cortex and hippocampus, accounting for the majority of expression changes. This is consistent with what one would expect according to neuroanatomical studies of depression and suicide and is also consistent with previous studies looking at discrete brain regions [4], [57]–[63]. The functional analysis using gene ontologies also revealed that an over-representation of genes involved in cell communication processes were globally altered. More specifically, among genes involved in synaptic transmission, a striking number of GABAergic receptor subunit genes were generally up-regulated among the suicides with major depression, but showed lower expression levels among the 2 other groups. We also observed for the suicide with major depression group a general up-regulation of AMPA receptors subunit genes and a global down-regulation of GRM3 receptors and glutamine synthase (GLUL) gene expression. Our study suggests the presence of consistent alterations of several genes coding for components of the same pathways across different brain regions.

The HG-133AB chipset contains around 44,000 probe sets many of which may not be expressed at biologically significant or detectable levels. Accordingly, Jongeneel et al. estimated that between 10 to 15 thousand transcripts are actually expressed in several types of human cell lines [64]. For that reason and in order to reduce the multiplicity problem, we used a combination of filtering methods in order to include in our analysis only transcripts that were actually expressed and reliably detectable. This approach efficiently allows to significantly reduce the total number of analyzed probe sets without notably decreasing the number of truly positive genes [65]. This resulted in an average of around 15,000 probe sets analyzed per region Second, in order to control for type I errors, we also used a combination of stringent *P*-value thresholds (≤0.01 both at the ANOVA and post-hoc test), as well as a fold change of at least 1.3 in either direction. Most importantly, by focusing on results that replicate across several different brain regions, which constitute partially independent experiments, we are likely to have significantly reduced the occurrence of type I errors in our study.

The current approach led to the identification of 4,472 differentially expressed probe sets over the 17 brain regions (Table 2). As expected, and in accordance with the neuroanatomical and post-mortem biomarkers literature, three prefrontal cortex areas, BA8,9, BA10, BA46, and the hippocampus, had the highest number of differentially expressed probe sets, thus confirming the implication of these regions in the pathophysiology of suicide and major depression (Table 2 and Figure 1). These four areas have been well characterized and have been previously shown to be implicated in suicidal behaviors and depression in numerous post-mortem studies [4], [66]–[78]. In vivo, neuroimaging studies have also pointed to alterations in the prefrontal cortex and in the hippocampus in patients suffering from major depression [29], [59], [61], [79]–[81]. Our study provides on a genomic scale, potential molecular targets that may account for those alterations in the brains of suicide victims with and without major depression.

Functional analysis using gene ontologies [82] was performed across the 17 regions using a new tool (ErmineJ) that efficiently addresses many of the limitations and problems of the initial gene ontology tools [83], by implementing more comprehensive algorithms and the possibility of performing analyses in parallel. This global functional ontological profiling revealed specific ontological categories commonly overrepresented in all the regions investigated in this study, and further investigation showed that an important proportion of genes belonged to cell-communication processes. Among these, a remarkable number of probe sets corresponded to genes coding for various molecular units of the GABAergic and glutamatergic neurotransmitter systems.

L-glutamic acid (glutamate) and GABA are respectively the main excitatory and inhibitory neurotransmitters in the central nervous system [84]. Growing evidence has supported alterations in both of these neurotransmitter systems in major depression [22], [23], [53]–[55] and suicide [17], [19], [20], [55], [56]. Sanacora et al. [21] using a magnetic resonance spectroscopy protocol observed elevated levels of glutamate and lower levels of GABA in the occipital cortex of subjects diagnosed with major depression. Furthermore, Hasler et al. [85] demonstrated that abnormal reductions in glutamate/glutamine and GABA concentrations are present in the prefrontal cortex of unmedicated depressed patients. Our results are also in concordance with those of Choudary et al. [18], who performed a gene expression study in the cingulate and prefrontal cortex brain areas of suicides and depressed suicides using one of the chips (HG-U133A) of the microarray set used in our study. Interestingly, their results point to similar alterations in glutamate recycling (glutamine synthase, GLUL), glutamate receptors (GRIA1, GRIA3, GRIK1, GRM3) and GABA receptors (GABARB3, GABRD, GABARG2) in depressed suicides versus controls. Also, recently, Merali et al. [55] observed altered levels of GABA(A) receptor subunits (α1, α3, α4 and δ) in the BA10 of depressed suicide victims versus non-depressed controls.

GRIA3, which was also confirmed to be differentially expressed by SemiQ RT-PCR (Table 5), is of particular interest in suicide as it was significantly down-regulated in the prefrontal cortex in both suicide groups (BA46, Figure 4), with and without major depression, suggesting an implication in suicide irrespective of the presence of major depression. This result is particularly important in the light of the recent observation by Laje et al. [86] that genetic variation at the GRIA3 gene seems to be associated with suicidal ideation during citalopram therapy and suggests that expression changes in this gene may also confer susceptibility to suicide and suicidal ideation in antidepressant treated patients.

Glia and astroglia in particular are responsible for the uptake,via the glial glutamate transporter (EAAT2) and metabolism and recycling, via glutamine synthase (GLUL) of glutamate [87]. Glutamine synthase is responsible for the recycling of glutamate by its conversion into glutamine, which is then released by the astrocytes and taken up at the synaptic terminals where it can be reconverted into glutamate or GABA [87]. Glutamine synthase was down-regulated in several prefrontal and parietal areas of brains of suicides with major depression, but not in suicides without major depression suggesting a depression specific dysregulation of glutamate recycling probably leading to altered glutamatergic and/or GABAergic neurotransmission. At the same time the majority of ionotropic glutamatergic receptors differentially expressed were up-regulated in these brain regions in depressed suicides, reinforcing the idea of a substantial alteration of glutamatergic neurotransmission in this group. Given the importance of some of these molecules in glial metabolism, and the growing evidence pointing to astroglial alterations in major depression [87], future studies should investigate cell specific changes in gene expression by means of laser capture microdissection in the brains of depressed suicides.

This hypothesis, if true, is in agreement with the observation that a single dose of Ketamine, an NMDA antagonist, is sufficient to produce a rapid and long lasting antidepressant effect [88]. Glutamate seems to mediate stress-induced neuronal atrophy in the hippocampus [89]. In addition, although not always consistent, there are different lines of evidence, comprising peripheral studies [90], postmortem brain studies [20], and in-vivo imaging studies [91] reporting glutamatergic dysfunction in major depression. Interestingly, glutamatergic neurotransmission is closely controlled by intracellular levels of polyamines, spermine and spermidine being specific modulators of NMDA and AMPA receptors activity [92]–[97]. Polyamines, and more specifically SSAT, the rate limiting enzyme in the catabolism of polyamines, were associated with suicide and depression in a previous study by our group [16]. Polyamines modulate GABAergic and glutamatergic neurotransmission, genes of those systems as well as SSAT were also found to be altered in the present study. In light of these observations, it is important to consider the polyamine-glutamatergic systems as a possible target for future strategies for the treatment of major depression.

Serotonergic and adrenergic dysfunction has been implicated in suicide [5], [70]_,_ yet we did not detect a significant representation of genes coding for components of these neurotransmitter systems in our differential expression analyses. This result is in accordance with all other microarryay experiments performed to date using suicide brains, where no serotonergic genes have been detected as differentially expressed [16], [38].

While our microarray experiment sampled multiple brain regions making our analysis global in nature, although enriched for frontal cortical regions due to the implication of these regions in depression and suicide, not all differentially expressed GABAergic and glutamatergic genes were differentially expressed across all regions. In general, we observed a particular probe set as differentially expressed across 2–3 regions, many of which did not overlap with other probe sets identified as differentially expressed. Still, the consistency of the dysregulation in the GABA-glutamate gene systems was striking.

Even though possible limitations regarding pre- and post-mortem factors, such as agonal period, alcohol abuse/dependence and post mortem interval were experimentally controlled for in this study, the conclusions presented here are to be taken with caution and need to be confirmed in an independent and larger sample. Our study design does not allow to clearly differentiating the alterations solely related to suicide from those specific to major depression. This would be possible to resolve only by including a group of matching patients with major depression who did not die by suicide, but such a group would be too difficult to obtain due to the demographic characteristics of suicide victims and depressed patients. Nevertheless, our results are interesting as they shed light into the molecular alterations simultaneously taking place in several important brain regions of individuals with and without major depressive disorder at the moment of their suicide. Another limitation of this study is the choice of 1.3 as a fold-change cut-off. While this allowed us to focus on more robust effects, it prevented us from detecting more subtle changes in gene expression that may be at play.

In conclusion, this is, to our knowledge, the first study attempting to determine global brain expression changes taking place in the brain of suicide victims with and without major depression. We observed global changes in genes implicated in synaptic transmission, and more specifically, in genes involved in GABAergic (inhibitory) and glutamatergic (excitatory) neurotransmission. Further studies are warranted in order to examine in detail the cellular origin of the alterations observed in our analyses, to validate the observed changes using complementary approaches and to investigate possible genetic factors related to the observed alterations.

## Supporting Information

Table S1 Genes differentially expressed after an ANCOVA analysis between the three groups with substance history or presence in the toxicological screening as a covariate. The 17 regions were analyzed, only genes associated with substance use are shown the significant genes at the P<0.01 level.
(0.07 MB DOC)

Table S2 The effect of substances on the expression of glutamatergic and GABAergic genes.
(0.04 MB DOC)

Table S3 Q-RT-PCR results from acute (N = 10) and chronic (N = 15) alcohol experiments in rats.
(0.03 MB DOC)

Table S4 Glutamatergic and gabaergic raw data
(0.61 MB XLS)
