# Supplementary material for: Global Brain Gene Expression Analysis Links Glutamatergic and GABAergic Alterations to Suicide and Major Depression
Source: PLoS One. 2009 Aug 11;4(8):e6585. doi: 10.1371/journal.pone.0006585 (PMC2719799; doi:10.1371/journal.pone.0006585)
Supplement: Table S1 — Genes differentially expressed after an ANCOVA analysis between the three groups with substance history or presence in the toxicological screening as a covariate. The 17 regions were analyzed, only genes associated with substance use are shown the significant genes at the P<0.01 level. (0.07 MB DOC) [file pone.0006585.s001.doc]

Table S1: Genes differentially expressed after an ANCOVA analysis between the three groups with substance history or presence in the toxicological screening as a covariate. The 17 regions were analyzed, only genes associated with substance use are shown the significant genes at the *P*<0.01 level.

| Region | Probe set | Sequence Clusters: Cluster Title | Gene Symbol | *P* (Group) | F (Group) | *P* (Substance) | F (Substance) |
| --- | --- | --- | --- | --- | --- | --- | --- |
| Amy | 226406_at | Chromosome 18 open reading frame 25 | C18orf25 | 0.012 | 5.3 | 0.001 | 16.0 |
| BA11 | 205018_s_at | muscleblind-like 2 (Drosophila) | MBNL2 | 0.001 | 11.2 | 0.006 | 9.8 |
| BA11 | 208771_s_at | leukotriene A4 hydrolase | LTA4H | 0.001 | 10.1 | 0.003 | 12.0 |
| BA11 | 226025_at | ankyrin repeat domain 28 | ANKRD28 | 0.000 | 18.4 | 0.006 | 10.2 |
| BA29 | 205846_at | protein tyrosine phosphatase, receptor type, B | PTPRB | 0.008 | 6.2 | 0.009 | 8.2 |
| BA4 | 208631_s_at | hydroxyacyl-Coenzyme A dehydrogenase/3-ketoacyl-Coenzyme A thiolase/enoyl-Coenzyme A hydratase (trifunctional protein), alpha subunit | HADHA | 0.001 | 11.6 | 0.006 | 10.4 |
| BA4 | 225308_s_at | KIAA1728 protein | KIAA1728 | 0.000 | 13.5 | 0.004 | 11.3 |
| BA44 | 222651_s_at | trichorhinophalangeal syndrome I | TRPS1 | 0.000 | 10.8 | 0.005 | 9.3 |
| BA46 | 204719_at | ATP-binding cassette, sub-family A (ABC1), member 8 | ABCA8 | 0.006 | 6.5 | 0.009 | 8.3 |
| BA46 | 203275_at | Interferon regulatory factor 2 | IRF2 | 0.000 | 16.1 | 0.001 | 14.4 |
| BA46 | 227987_at | Vacuolar protein sorting 13A (yeast) | VPS13A | 0.023 | 4.5 | 0.008 | 8.6 |
| BA46 | 226538_at | Mannosidase, alpha, class 2A, member 1 | MAN2A1 | 0.016 | 5.0 | 0.005 | 9.5 |
| BA46 | 225129_at | Copine II | CPNE2 | 0.003 | 7.8 | 0.002 | 12.9 |
| BA46 | 201552_at | Lysosomal-associated membrane protein 1 | LAMP1 | 0.004 | 7.4 | 0.009 | 8.2 |
| BA46 | 227461_at | Stonin 2 | STN2 | 0.000 | 14.2 | 0.010 | 8.0 |
| BA46 | 226252_at | CDNA FLJ34585 fis, clone KIDNE2008758 |  | 0.000 | 11.1 | 0.009 | 8.2 |
| BA46 | 224909_s_at | Phosphatidylinositol 3,4,5-trisphosphate-dependent RAC exchanger 1 | PREX1 | 0.000 | 11.0 | 0.006 | 9.4 |
| BA46 | 226490_at | NHS-like 1 | NHSL1 | 0.001 | 10.1 | 0.002 | 12.0 |
| BA46 | 217976_s_at | Dynein, cytoplasmic, light intermediate polypeptide 1 | DNCLI1 | 0.001 | 10.8 | 0.001 | 14.1 |
| BA46 | 233881_s_at | Toll interacting protein | TOLLIP | 0.001 | 10.9 | 0.007 | 8.9 |
| BA8,9 | 214663_at | dusty protein kinase | DustyPK | 0.000 | 18.1 | 0.008 | 9.3 |
| BA8,9 | 241079_at | pumilio homolog 1 (Drosophila) | PUM1 | 0.001 | 11.1 | 0.009 | 8.8 |
| BA8,9 | 219945_at | DEAD (Asp-Glu-Ala-Asp) box polypeptide 25 | DDX25 | 0.000 | 14.3 | 0.004 | 11.3 |
| BA8,9 | 202208_s_at | ADP-ribosylation factor-like 7 | ARL7 | 0.001 | 10.5 | 0.009 | 8.9 |
| Hippo | 226567_at | ubiquitin specific protease 14 (tRNA-guanine transglycosylase) | USP14 | 0.003 | 8.2 | 0.008 | 8.8 |
| Hippo | 243179_at |  |  | 0.000 | 16.4 | 0.001 | 17.7 |
| Hippo | 220367_s_at | mSin3A-associated protein 130 | SAP130 | 0.002 | 9.2 | 0.006 | 9.6 |
| NAcc | 207248_at | potassium voltage-gated channel, shaker-related subfamily, member 4 | KCNA4 | 0.000 | 13.7 | 0.008 | 8.7 |
