# Supplementary material for: Global Brain Gene Expression Analysis Links Glutamatergic and GABAergic Alterations to Suicide and Major Depression
Source: PLoS One. 2009 Aug 11;4(8):e6585. doi: 10.1371/journal.pone.0006585 (PMC2719799; doi:10.1371/journal.pone.0006585)
Supplement: Table S2 — The effect of substances on the expression of glutamatergic and GABAergic genes. (0.04 MB DOC) [file pone.0006585.s002.doc]

Table S2: The effect of substances on the expression of glutamatergic and GABAergic genes.

| **Probeset ID** | **Gene Symbol** | **Gene Title** | **p-value**  **Diagnosis** | **p-value**  **subst Dx or Hx** | **p-value**  **Diagnosis * subst Dx or Hx** | **GFoldChange**  **No vs Yes** |
| --- | --- | --- | --- | --- | --- | --- |
| **208869_s_at** | GABARAPL1 | GABA(A) receptor-associated protein like 1 | 0.097579 | 0.354054 | 0.660083 | 1.10381 |
| **208868_s_at** | GABARAPL1 | GABA(A) receptor-associated protein like 1 | 0.218323 | 0.665678 | 0.994929 | -1.02931 |
| **206849_at** | GABRG2 | gamma-aminobutyric acid (GABA) A receptor, gamma 2 | 0.183426 | 0.857953 | 0.637982 | 1.03662 |
| **206525_at** | GABRR1 | gamma-aminobutyric acid (GABA) receptor, rho 1 | 0.836325 | 0.598689 | 0.891552 | -1.01654 |
| **203159_at** | GLS | glutaminase | 0.157824 | 0.827927 | 0.29785 | 1.01907 |
| **203157_s_at** | GLS | glutaminase | 0.168299 | 0.412436 | 0.387254 | 1.11925 |
| **203158_s_at** | GLS | glutaminase | 0.195782 | 0.792406 | 0.504283 | 1.04755 |
| **221510_s_at** | GLS | glutaminase | 0.595529 | 0.385606 | 0.254418 | 1.01961 |
| **211414_at** | GLS | glutaminase | 0.853263 | 0.996783 | 0.461644 | 1.0001 |
| **200648_s_at** | GLUL | glutamate-ammonia ligase (glutamine synthetase) | 0.119887 | 0.841489 | 0.390718 | 1.03695 |
| **217202_s_at** | GLUL | glutamate-ammonia ligase (glutamine synthetase) | 0.142732 | 0.68421 | 0.597834 | 1.07906 |
| **215001_s_at** | GLUL | glutamate-ammonia ligase (glutamine synthetase) | 0.168822 | 0.950139 | 0.434704 | 1.00974 |
| **211520_s_at** | GRIA1 | glutamate receptor, ionotropic,  AMPA 1 | 0.049351 | 0.513371 | 0.928259 | 1.02912 |
| **215634_at** | GRIA1 | Glutamate receptor, ionotropic, AMPA 1 | 0.123684 | 0.05492 | 0.086167 | 1.10199 |
| **208032_s_at** | GRIA3 | glutamate receptor, ionotrophic, AMPA 3 | 0.284547 | 0.871411 | 0.879784 | -1.00909 |
| **217565_at** | GRIA3 | glutamate receptor, ionotrophic, AMPA 3 | 0.609367 | 0.579744 | 0.615095 | -1.03068 |
| **205814_at** | GRM3 | glutamate receptor, metabotropic 3 | 0.885416 | 0.32644 | 0.831333 | 1.10071 |
| **205152_at** | SLC6A1 | solute carrier family 6(neurotransmitter transporter, GABA), member 1 | 0.888987 | 0.40172 | 0.327462 | 1.06555 |
