# Supplementary material for: Global Brain Gene Expression Analysis Links Glutamatergic and GABAergic Alterations to Suicide and Major Depression
Source: PLoS One. 2009 Aug 11;4(8):e6585. doi: 10.1371/journal.pone.0006585 (PMC2719799; doi:10.1371/journal.pone.0006585)
Supplement: Table S3 — Q-RT-PCR results from acute (N = 10) and chronic (N = 15) alcohol experiments in rats. (0.03 MB DOC) [file pone.0006585.s003.doc]

Table S3: Q-RT-PCR results from acute (N=10) and chronic (N=15) alcohol experiments in rats.

| Gene | Chronic (F, p) | Acute (T, p) |
| --- | --- | --- |
| GABARAPL1 | 2.83, 0.10 | 0.98, 0.36 |
| GABARD | 0.11, 0.90 | 0.43, 0.68 |
| GABARG1 | 0.37, 0.70 | 0.91, 0.39 |
| GABRG2 | 0.60, 0.56 | 0.82, 0.44 |
| GABRR1 | 0.11, 0.90 | 0.75, 0.48 |
| GLS | 0.40, 0.68 | 0.95, 0.37 |
| GLUL | 0.01, 0.99 | 1.6, 0.15 |
| GRIA1 | 1.74, 0.22 | 1.28, 0.24 |
| GRIA3 | 2.16, 0.16 | 1.91, 0.09 |
| GRM3 | 1.04, 0.39 | 1.10, 0.31 |
| SLC6A1 | 0.96, 0.41 | 0.15, 0.88 |
